# Effect of an antioxidant supplement containing high dose lutein and zeaxanthin on macular pigment and skin carotenoid levels

**DOI:** 10.1038/s41598-020-66962-2

**Published:** 2020-06-24

**Authors:** Akira Obana, Yuko Gohto, Risa Nakazawa, Takanobu Moriyama, Werner Gellermann, Paul S. Bernstein

**Affiliations:** 1 Department of Ophthalmology, Seirei Hamamatsu General Hospital, Hamamatsu, Shizuoka Japan; 2 Hamamatsu BioPhotonics Innovation Chair, Institute for Medical Photonics Research, Preeminent Medical Photonics Education & Research Center, Hamamatsu University School of Medicine, Hamamatsu, Shizuoka Japan; 3 Longevity Link Corporation, Salt Lake City, UT United States of America; 4 Department of Ophthalmology and Visual Sciences, Moran Eye Center, University of Utah School of Medicine, Salt Lake City, Utah United States of America

**Keywords:** Health care, Medical research

## Abstract

The effect of a high dose lutein/zeaxanthin supplement on macular pigment optical density (MPOD) and skin carotenoid (SC) levels in healthy subjects was investigated. This is a prospective, single-arm, open-label study. Subjects were 16 Japanese, age 26–57 years. Subjects took a supplement containing 20 mg/day of lutein, 4 mg/day of zeaxanthin, and other antioxidants (vitamin C, vitamin E, zinc, copper) for 16 weeks. MPOD levels were measured by a two-wavelength autofluorescence imaging technique. SC levels were measured by reflection spectroscopy. Total volume of MPOD within 9° eccentricity significantly increased by week 8 and continued to increase until week 16 (*p* < 0.0001, two-way factorial ANOVA). The increase rate of MPOD was significantly higher in subjects with body mass index (BMI) less than 25 kg/m^2^ (n = 13) compared to those of 25 kg/m^2^ and higher (n = 3). SC levels increased significantly by week 4 and continued to increase until week 16 (*p* < 0.0001, two-way factorial ANOVA). All subjects completed the study without any serious adverse events. These results demonstrated the effectiveness of a high dose lutein/zeaxanthin supplement for MPOD volume and SC levels without serious adverse events.

## Introduction

The human macula contains yellow pigment, called macular pigment (MP), consisting of three carotenoids, lutein ((3R, 3′R, 6′R) -lutein), zeaxanthin ((3R, 3′R) -zeaxanthin), and *meso*-zeaxanthin ((3R, 3′S; *meso*) –zeaxanthin)^1,2^. MP absorbs blue light and acts as a filter that may attenuate photochemical damage to the retina due to blue light exposure and potentially protects against light-induced oxidative damage in the retina by quenching oxygen radicals^3–5^. MP functions to improve contrast sensitivity and reduces night glare^6–9^. Some studies have reported that MP optical density (MPOD) levels in eyes with age-related macular degeneration (AMD) are significantly lower than those in normal, healthy eyes^10,11^. The light protection effect of MP is thought to prevent age-related macular degeneration (AMD)^7,12–15^, and our previous study on a Japanese population indicated that lower MPOD levels may be a risk factor for AMD progression^16^.

Carotenoids such as lycopene, alpha-. beta-, gamma-, delta-carotene, beta-cryptoxanthin, lutein, and zeaxanthin are contained in the epidermis, dermis, and subcutaneous fat^17^. These carotenoids protect skin against oxidation induced by sunlight exposure. Lutein and zeaxanthin have been reported to reduce lipid peroxidation and increase moisture in the skin^18^. The anti-oxidative effect of lutein also protects against UV-induced skin damage^19^. Regarding the subset of carotenoids found in the human macula, previous studies have reported a weak to moderate correlation between MPOD and skin carotenoid (SC) levels measured by resonance Raman spectroscopy (RRS)^20–23^.

The Age-related Eye Disease Study (AREDS) Research Group conducted the first multi-center, randomized trial to verify the prophylactic effect of a supplement containing vitamin C, vitamin E, beta-carotene, and zinc, and demonstrated that this supplement formula reduced the 5-year risk of advanced AMD in persons at risk by 25%^24,25^. A second clinical trial was conducted to investigate the effect of lutein/zeaxanthin instead of beta-carotene^26^, because several observational studies demonstrated that higher dietary intake of lutein/zeaxanthin was associated with a decreased risk of advanced AMD^27^. According to the AREDS2 study, a prophylactic effect of lutein/zeaxanthin-containing anti-oxidative supplements can be achieved at least for the lowest dietary intake of lutein and zeaxanthin quintile^28^. The formula recommended by AREDS2 is: vitamin C 500 mg, vitamin E 400 IU, lutein 10 mg, zeaxanthin 2 mg, zinc oxide 80 mg, and cupric oxide 2 mg. Based on this formula, several commercial products have been released in Japan, although the actual amount of each ingredient may be modified by the manufacturer. For example, commercial products in Japan contain less zinc than the AREDS formula since the maximum allowable amount of zinc in Japan is 30 mg per day, whereas the AREDS formula contains 65 mg of zinc (80 mg of zinc oxide).

In this study, we investigated the change in MPOD and SC levels due to supplement intake of high doses of lutein/zeaxanthin in healthy subjects. This is the first study to show the effects of lutein and zeaxanthin supplementation with vitamin C, vitamin E, zinc, and copper on skin carotenoid levels.

## Results

This is a prospective, single-arm, open-label study at a single institute (Seirei Hamamatsu General Hospital). Thirty-six healthy volunteers were evaluated between June 2018 and May 2019, and twenty were excluded from enrollment due to incompatibility with the inclusion criteria (Table 1). Six had a spherical equivalent refractive error greater than −6.0 diopter. Thirteen subjects had MPOD exceeding 0.64 at 0.5° eccentricity by heterochromatic flicker photometry (Macular Metrics II Macular Metrics Inc., MA, USA, [abbreviated as HFP-MM II]). One had a choroidal nevus in one eye and MPOD exceeding 0.64 in the other eye. Of the remaining 16 subjects enrolled in the study, 2 were men and 14 were women, ranging in age from 26–57 years (mean age 46.3 ± 7.9). Demographic data of the subjects at baseline and rate of supplement intake during the study period (rate of supplement intake = number of days supplements were taken/total days of the study period) are shown in Table 2.

**Table 1:** Inclusion Criteria.

Japanese
Age: 20–60 years old
No ocular pathologies detected by slit-lamp biomicroscopy and fundus ophthalmoscopy
Visual acuity of 0.8* or above at the time of MPOD measurement
Spherical equivalent refractive error less than −6.0 diopter
No gastrointestinal diseases that could interfere with dietary absorption
No diabetes
No history of lutein/zeaxanthin or co-antioxidants supplement intake
No allergies to lutein/zeaxanthin
Not pregnant or breast-feeding
Pupil diameter of 6.5 mm or more by mydriatic agent.
MPOD at 0.5° eccentricity measured by MMII is 0.64 or less.

*The visual acuity was measured using a decimal visual acuity test chart. 0.8 is equivalent to 20/25 of Snellen visual acuity or 0.097 of logMAR.

MPOD: macular pigment optical density; MMII: Macular Metrics II.

**Table 2:** Demographic data at baseline and rate of supplement intake at the end of the study for all subjects.

Subject	Sex	Age (year)	Body mass index (kg/m^2^)	Study Eye	Intraocular pressure (mmHg)	Spherical equivalent refractive error (Diopter)	Central macular thickness by OCT (μm)	Rate of supplement taking (%)
1	female	49	21.5	Right	11.7	−3.25	246	94
2	female	48	29.0	Right	19.0	0.00	283	100
3	female	40	22.9	Right	18.7	−5.00	239	96
4	female	54	20.3	Left	13.3	−3.00	257	92
5	female	42	21.1	Left	12.3	−4.75	263	100
6	female	41	20.8	Right	18.3	0.00	272	98
7	female	44	21.1	Left	11.7	0.00	263	99
8	female	57	28.1	Right	17.7	−0.75	254	97
9	female	51	19.7	Right	17.0	−1.00	265	100
10	female	51	19.8	Left	12.0	−1.00	264	91
11	female	44	16.8	Left	14.3	−2.50	264	96
12	male	38	22.6	Right	16.3	−3.50	253	88
13	female	26	23.5	Left	18.3	−0.50	258	100
14	female	53	21.2	Left	10.0	−1.50	246	99
15	male	46	26.1	Left	20.3	−3.75	283	94
16	female	56	19.6	Left	14.0	−0.75	239	100

Rate of supplement taking = actual no. of days of intake/total no. of days of the study period.

### Change in visual function and OCT imaging

Best corrected far and near decimal visual acuities at baseline were 1.2 and 1.0 in all subjects, and no change in visual acuity throughout the study period was observed in any subject. Mean retinal thickness at 1-mm diameter central area (central retinal thickness, CRT) measured by optical coherence tomography (OCT) of each subject at baseline was between 239–283 μm. Mean CRT values ± standard deviation (SD) for all subjects at baseline, week 4, week 8, week 12, and week 16 were 259.2 ± 10.5, 258.4 ± 11.4, 259.2 ± 12.3, 259.9 ± 13.0, and 259.1 ± 11.1 μm, respectively. No significant differences in CRT were observed at each time period for all subjects (*p* = 0.661, two-way factorial ANOVA). Baseline contrast sensitivity (C_AULCSF [the area under the log contrast sensitivity function]) was from 1.61 to 1.84, and glare disability (G_AULCSF) was from 0.85 to 1.49. Mean C_AULCSF at baseline, week 8, and week 16 were 1.74 ± 0.07, 1.76 ± 0.11 and 1.76 ± 0.08, respectively. Mean G_AULCSF at baseline, week 8, and week 16 were 1.25 ± 0.15, 1.26 ± 0.12, and 1.28 ± 0.13, respectively. No significant differences in C_AULCSF and G_AULCSF were observed at each time period for all subjects (*p* = 0.567, 0.577, two-way factorial ANOVA).

### Changes in MPOD levels

**Figure 1:**
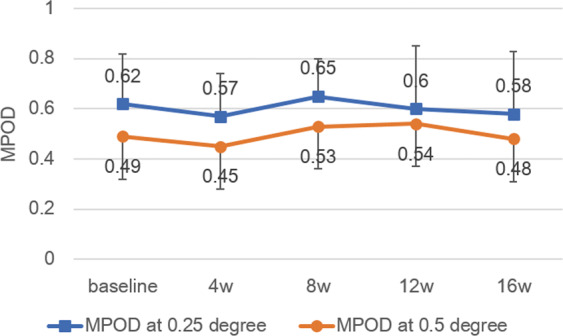
Changes in mean MPOD measured by Macular Metrics II. No significant change in mean macular pigment optical density (MPOD) at both eccentricities at each time period (0.25°, *p* = 0.458, 0.5°, *p* = 0.056, two-way factorial ANOVA). Blue: 0.25°; Red: 0.5°. Bar represents standard deviation.

MPOD levels of the study eye were measured using two different methods at baseline and 4, 8, 12, and 16 weeks after starting supplement intake. MPOD levels measured by HFP-MMII at baseline varied between subjects from 0.31–1.10 at 0.25° eccentricity and 0.27–0.63 at 0.5° eccentricity. Mean values at 0.25° and 0.5° eccentricities were 0.62 ± 0.20 and 0.49 ± 0.11, respectively. Mean baseline MPOD levels and mean MPOD levels obtained for all subjects at time points 4,8,12, and 16 weeks are shown in Fig. 1. No significant differences in mean MPOD at either eccentricity at each time period (*p* = 0458, 0.056, two-way factorial ANOVA) were observed.

**Figure 2:**
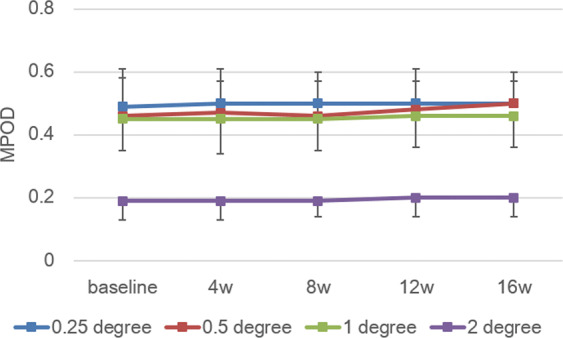
Changes in mean local MPOD measured by SPECTRALIS. No significant change in mean MPOD at all four eccentricities during the study period. Blue: 0.25°; Red: 0.5°; Green: 1°, Purple: 2°. Bar represents standard deviation.

**Figure 3:**
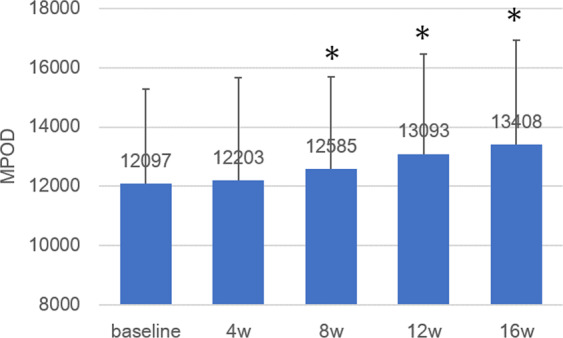
Change in mean total MPOV measured by SPECTRALIS. Mean total macular pigment optical density volume (MPOV) at week 8, 12, and 16 are significantly higher than that at baseline (*p* < 0.0001, two-way factorial ANOVA, baseline/week 8, *p* = 0.034; baseline/week12, *p* < 0.0001; baseline/week 16, *p* < 0.0001, post-hoc with LSD). Bar represents standard deviation.

Local MPOD of each subject measured by two-wavelength autofluorescence imaging, AFI-SP (autofluorescence imaging, a prototype SPECTRALIS MultiColor platform with MPOD module [Heidelberg Engineering, Heidelberg, Germany]) at 0.25°, 0.5°, 1°, and 2° eccentricities at baseline were 0.28–0.73, 0.19–0.64, 0.20–0.60, and 0.05–0.28, respectively. Mean values at 0.25°, 0.5°, 1°, and 2° eccentricities were 0.49 ± 0.12, 0.46 ± 0.12, 0.45 ± 0.10, and 0.19 ± 0.06, respectively. Corresponding mean MPOD levels obtained for all subjects at baseline and time points 4,8,12, and 16 weeks are shown in Fig. 2. No significant difference in local MPOD levels for each subject during the test period were observed again for the four eccentricities (*p* = 0.801, 0.310, 0.205, 0.534, two-way factorial ANOVA). Total MPOV for each subject at baseline ranged from 4527 to 16535. Mean values at baseline, week 4, week 8, week 12 and week 16 were 12097 ± 3192, 12203 ± 3476, 12585 ± 3111, 13093 ± 3371, and 13408 ± 3521, respectively. Corresponding mean total MPOV levels obtained for all subjects at baseline and time points 4,8,12, and 16 weeks are shown in Fig. 3. The mean total MPOV at week 4 was not significantly higher than at baseline. However, those at week 8, 12, and 16 were significantly higher than at baseline (*p* < 0.0001, two-way factorial ANOVA, baseline/week 4, *p* = 0.641, baseline/week 8, *p* = 0.034; baseline/week12, *p* < 0.001; baseline/week 16, *p* < 0.001, post-hoc with LSD). The increase in total MPOV at week 16 for each subject (i.e. (total MPOV at week 16 – total MPOV at baseline) / total MPOV at baseline) ranged between 3–27%, with a mean of 11%.

A significant negative correlation between body mass index (BMI) at baseline and increase in local MPOD levels at week 16, excluding local MPOD at 0.5°, was observed (Table 3). The correlation between BMI and increased local MPOD at 0.5° was marginal, however. Subjects with higher BMI showed a smaller increase of local MPOD levels than subjects with lower BMI. No significant correlation between BMI and the increase of total MPOV at week 16 was observed. However, when BMIs were divided into two groups, one with BMI lower than 25 kg/m^2^ (n = 13), and the other with BMI 25 kg/m^2^ and higher that is defined as obese in Japanese (n = 3), a significant difference in the increase of local MPOD at 0.25°, 1°, and 2° and total MPOV at week 16 was observed (Table 4). Percentage increases were higher in the lower BMI group. Local MPOD and total MPOV did not increase in the higher BMI group.

**Table 3:** Correlation between body mass index and rate of increase for local MPOD or total MPOV at week 16.

	Pearson’s correlation coefficient	*p*
**MPOD at 0.25°**	**−0.629**	**0.009**
MPOD at 0.5°	−0.474	0.064
**MPOD at 1°**	**−0.622**	**0.010**
**MPOD at 2°**	**−0.567**	**0.022**
Total MPOV	−0.338	0.200

MPOD: macular pigment optical density; MPOV macular pigment optical volume.

**Table 4:** The rate of increase for local MPOD and total MPOV at week 16 in subjects with BMI* less than 25 and subjects with BMI 25 and more.

	BMI less than 25(n = 13)	BMI 25 and more(n = 3)	*p* (t-test)
MPOD at 0.25°	**0.005**	**−0.10**	**0.001**
MPOD at 0.5°	0.26	−0.07	0.344
MPOD at 1°	**0.04**	**−0.05**	**0.001**
MPOD at 2°	**0.04**	**−0.07**	**0.001**
Total MPOV	**0.13**	**−0.12**	**0.007**

MPOD: macular pigment optical density; MPOV macular pigment optical volume; BMI: body mass index.

### Changes in skin carotenoid levels

**Figure 4:**
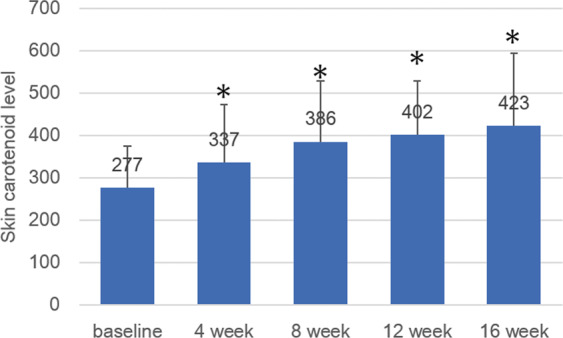
Change in mean skin carotenoid levels of all subjects.Mean SC levels at week 4, 8, 12, and 16 are significantly higher than that at baseline (*p* < 0.0001, two-way factorial ANOVA, baseline/week 4, *p* = 0.010; baseline/week 8, *p* < 0.0001; baseline/week12, *p* < 0.0001; baseline/week 16, *p* < 0.0001, post-hoc with LSD). Bar represents standard deviation.

SC levels of each subject at baseline ranged from 37–483. Mean SC levels of all subjects at baseline, week 4, week 8, week 12, and week 16 were 277 ± 98, 337 ± 137, 380 ± 144, 402 ± 128, and 423 ± 172. The change in mean SC levels of all subjects is shown in Fig. 4. Mean SC levels at week 4, 8, 12, and 16 were significantly higher than that at baseline (*p* < 0.0001, two-way factorial ANOVA, baseline/week 4, *p* = 0.010; baseline/week 8, *p* < 0.0001; baseline/week12, *p* < 0.0001; baseline/week 16, *p* < 0.0001, post-hoc with LSD). Percentage increases in SC levels at week 16 of each subject except for one, (SC level at week 16 – SC level at baseline) / SC level at baseline) ranged between 16–114%, with a mean of 48%. For an exceptional subject, discussed in the discussion section who scored a noticeably low SC value at baseline, the increase in SC amounted to 616%. No significant correlation between BMI at baseline and the increase in SC levels at week 16 was observed (Pearson’s coefficient: 0.126, *p* = 0.641). In addition, no significant difference in the increased SC levels at week 16 between subjects in the low and high BMI groups was observed (*p* = 0.775, t-test).

### Mutual correlations

#### Correlation between contrast/glare sensitivity and MPOD levels / skin carotenoid levels at baseline

No significant correlation between C_AULCSF at baseline and SC levels, local MPOD levels, or MPOD volume were observed (Table 5). In addition, there were no significant correlations between G_AULCSF at baseline and SC levels, local MPOD levels, except for local MPOD levels at 0.98°, or MPOD volume (Table 5).

**Table 5:** Correlation coefficient between contrast/glare sensitivity and MPOD levels/skin carotenoid levels at baseline.

Contrast/glare sensitivity	MPOD/skin carotenoid levels	Pearson’s correlation coefficient	*p*
C_AULCSF	MPOD at 0.25°	−0.090	0.741
	MPOD at 0.5°	−0.345	0.190
	MPOD at 1°	−0.372	0.155
	MPOD at 2°	−0.243	0.364
	Total MPOV	−0.241	0.368
	Skin carotenoid levels	0.165	0.541
G_AULCSF	MPOD at 0.25°	−0.310	0.243
	MPOD at 0.5°	−0.348	0.186
	MPOD at 1°	**−0.504**	**0.046**
	MPOD at 2°	−0.367	0.162
	Total MPOV	−0.269	0.314
	Skin carotenoid levels	0.121	0.655

C_AULCSF: contrast the area under the log contrast sensitivity function; G_AULCSF: glare the area under the log contrast sensitivity function; MPOD: macular pigment optical density; MPOV macular pigment optical volume.

#### Correlation between MPOD levels and skin carotenoid levels

No significant correlation between SC levels and local MPOD levels or total MPOV at baseline were observed (Table 6).

**Table 6:** Correlation between MPOD levels and skin carotenoid levels at baseline.

	Pearson’s correlation coefficient	*p*
MPOD at 0.25°	−0.120	0.658
MPOD at 0.5°	−0.062	0.819
MPOD at 1°	−0.204	0.449
MPOD at 2°	−0.008	0.976
total MPOV	0.091	0.738

MPOD: macular pigment optical density; MPOV macular pigment optical volume.

#### Safety

All subjects completed the study without serious adverse events. Seven subjects reported changes in general condition for some days during the study period (Table 7). No subjects consulted a doctor due to the changes, but three took anti-inflammatory and/or analgesic medicine for the common cold and/or a headache.

**Table 7:** Change in general condition in each subject.

Subject No.	Number of the days change in general condition was felt	Symptom	Self-estimated reasons
1	1	Headache	Shoulder stiffness
	1	Menstrual pain	
2	0		
3	0		
4	0		
5	1	Sneezing, runny nose	Common cold
6	0		
7	1	Headache	Shoulder stiffness
8*	4	Sore throat	Common cold
9	0		
10	0		
11	1	Pain at the base of a finger	Unknown
12*	1	Stomach ache	Unknown
	4	Sneeze, runny nose, fever	Common cold
13*	2	headache	Unknown
	4	Stomach ache, nausea	Stress
14	0		
15	0		
16	0		

*Subjects who declared taking medicine.

Changes in blood tests between baseline and week 16 are shown in Table 8. Statistically significant changes were observed in platelet, total protein, total cholesterol, low-density lipoprotein (LDL)-cholesterol, creatinine, alkaline phosphatase, aspartate aminotransferase, and alanine aminotransferase levels. However, no clinical significance was determined for platelet, total protein, creatinine, alkaline phosphatase, alanine aminotransferase, as values at week 16 were within standard levels or had improved over baseline. An increase in total cholesterol was observed in 12 subjects at week 16, and four of them showed an increase from a normal range to values exceeding the upper limit. In contrast, of the four subjects who had total cholesterol values exceeding the normal limit at baseline, a decrease was observed in three of them at week 16. An increase in LDL-cholesterol was observed in 11 subjects, of which an increase from normal range to values exceeding the upper limit was observed in four of them.

**Table 8:** Mean values of blood tests at baseline and week 16.

	Baseline	Week 16	*p* (t-test)
Red blood cells (RBC)/μL	456 × 10^4^	457 × 10^4^	0.911
White blood cells (WBC)/μL	6,625	6,313	0.288
Platelets (PLT)/μL	**31.2** × 10^4^	**29.3** × 10^4^	**0.005**
Hemoglobin (Hb) (g/dL)	13.5	13.5	0.929
Hematocrit (Ht) (%)	41.5	41.7	0.451
MCV (fL)	90.8	91.5	0.029
MCH (pg)	29.5	29.5	0.731
MCHC (g/dL)	32.4	32.2	0.665
Total protein (TP) (g/dL)	**7.4**	**7.3**	**0.020**
Total cholesterol (TC) (mg/dL)	**212**	**227**	**0.011**
HDL-cholesterol (mg/dL)	74	76	0.225
LDL-cholesterol (mg/dL)	**124**	**135**	**0.012**
Albumin(Alb) (g/dL)	4.5	4.4	0.590
Total bilirubin (T-Bil) (mg/dL)	0.45	0.56	0.006
Triglyceride (TG) (mg/dL)	102	95	0.366
Urea nitrogen (mg/dL)	13.2	14.0	0.120
Creatinine (Cr) (mg/dL)	**0.66**	**0.63**	**0.015**
Alkaline phosphatase (ALP) (U/L)	**194**	**211**	**0.016**
Aspartate aminotransferase (AST) (U/L)	**17.4**	**20.5**	**0.006**
Alanine aminotransferase (ALT) (U/L)	**15.7**	**20.3**	**0.043**
gamma-glutamyl transpeptidase (γ-GT) (U/L)	21	26	0.130
Lactic dehydrogenase (LD) (U/L)	169	168	0.766
Glucose (mg/dL)	94.9	98.5	0.141

All urine qualitative tests for pH, protein, glucose, urobilinogen, ketone body were negative for all subjects at baseline and week 16. Urine blood tests were positive for 6 female subjects at baseline, of which two were positive at week 16.

## Discussion

Mean total MPOV significantly increased at 8 weeks after the start of supplement intake and continued to increase until the end of supplement intake at week 16. Mean SC levels also increased at 4 weeks and continued to increase until week 16. A significantly higher increase in local MPODs and total MPOV at week 16 was observed in subjects with BMI less than 25 kg/m^2^ compared to subjects with BMI 25 kg/m^2^ and more. All subjects completed the study without serious adverse events. These results demonstrated the effectiveness of the present supplement on the eye and skin.

In this study, only those subjects were selected who had MPOD levels of 0.64 and lower at 0.5° eccentricity. The rationale for this selection is the avoidance of subjects with already saturated macular pigment, who would not be able to respond with a further increase upon supplementation^29–31^. The value of 0.64 was chosen based on our previous study^32^. HFP-MM II was used for screening because it can be carried out without mydriasis. According to our previous study, MPOD hardly increased in myopic subjects in response to supplementation^32^. Consequently, subjects with myopia of −6.0D or higher were excluded as well.

A significant increase in MPOD levels measured by heterochromatic flicker photometry at four weeks and later has already been reported in many previous studies^12,30–35^. Concerning the time at which a significant increase was observed, one study used a high dose *meso*-zeaxanthin supplement^36^ and reported a rapid increase of MPOD just two weeks later. In contrast, a relatively slow increase of MPOD (significant increase observed only at 12 weeks and after) was reported for a dietary investigation study with spinach and corn^37^. One study, using the same lutein product as in this study (20 mg lutein and 4 mg zeaxanthin) reported a significant increase at 8 weeks and after^38^, which is in agreement with this study. Concerning the increase in amount of MPOD level, previous studies reported it increases between 4–39%^12,30–36^. Mean increase of total MPOV at week 16 in this study was 11%. Speed and achievable percentage MPOD increases are affected by various factors, such as the type of lutein (i.e., free versus esterified), the amount of zeaxanthin and *meso*-zeaxanthin, the matrix of the formulation, and subject characteristics (e.g., age, sex, MPOD at baseline, smoking history and BMI). When interpreting the results, it is important to consider the respective method used to measure MPOD and evaluated parameters such as local MPOD or MPOV. Therefore, comparison of these results with previous studies is difficult. However, the MPOD increase in this study was relatively slow and the percentage increase was moderate compared to previous studies. Possible reasons for this are as follows. The mean total MPOV at baseline of the subjects in this study was higher than in previous studies. Total MPOV at baseline in the study by G-Gomez was 5094, with a reference point at 7°, and in the study by Nolan, et al., it was 6593, with a reference point at 8° ^39^. Mean baseline total MPOV in this study was 12097, with a reference point at 9°. This value was converted to 8138 with a reference point at 7°, when we re-calculated MPOV with this reference point. While subjects with a relatively low MPOD levels were selected, a potentially still high MPOD at baseline might be a reason for the moderate increase. Other possible reasons are subject characteristics other than baseline MPOD and unknown interactions with other nutrients.

The ability of the supplement to increase MPOD was influenced by BMI in this study. The increase in total MPOV at week 16 was significantly higher in subjects with BMI less than 25 kg/m^2^ than in subjects with BMI 25 kg/m^2^ and higher (Table 4). Interestingly, local MPOD levels and total MPOV did not increase with supplement intake in subjects with BMI 25 kg/m^2^ and more. An increase of 5% or more in total MPOV was observed in 12 subjects (subjects no. 1, 3, 4, 5, 6, 7, 9, 10, 12, 13, 14, 16 in Table 3) and was not observed in 4 subjects. Of the four subjects whose total MPOV did not increase, three (subject no. 2, 8, 15 in Table 2) had the highest BMI in this study (29.0, 28.1, 26.1) and one subject (subject no. 11) had the lowest BMI (16.8 kg/m^2^). High BMI was reported to be a factor in low MPOD levels in previous studies^37,40^. Adipose tissue and the retina competed for lutein^37^. A higher amount of body fat provides a larger “sink” for lutein, which makes less lutein available for other tissues. The subject with the lowest BMI (162 cm height, 44 kg body weight) may have some form of diet malabsorption.

In this study, the effect of supplement intake was observed for total MPOV but not for local MPOD levels. The reason for this discrepancy is thought to be as follows. The MP distribution is not symmetrical and can be roughly divided into four major patterns^41^. From this, local MPOD levels could possibly vary between eyes, even in cases where the total amount of MP is the same. The local MPOD level at discrete eccentricities does not always reflect the total amount of MP at the macula. In contrast, total MPOV provides an overall description of MP across the macula. Total MPOV by AFI-SP was reported to be more appropriate to evaluate the effect of supplementation, and total MPOV could be the standard value to assess MP^42^.

In our previous study^23^, the average SC level was 32% higher in subjects taking lutein supplements relative to non-supplementing subjects. The possibility to increase SC levels by beta-carotene, lycopene, and vegetable juice consumption was demonstrated by interventional studies^43–46^. However, no interventional study using lutein/zeaxanthin supplements has been performed to date. This study directly confirmed the effect of lutein/zeaxanthin supplementation on SC levels for the first time. It has been reported that lycopene and beta-carotene levels are predominant in skin, and the amount of lutein and zeaxanthin is much less than carotene^47^. However, a more recent study found a higher level, roughly 15%, amount of lutein and zeaxanthin in heel skin samples^48^, and the present results were consistent with this finding. Our previous study^23^ showed that BMI was a factor for a significant difference in SC levels, but in this study, no significant difference was observed. A possible reason for this discrepancy is the small number of subjects in this study.

A significant increase in SC levels at week 4 was observed, while in contrast, a significant increase in MPOV was observed only at week 8. The reason for this time lag is considered to be as follows. SC levels are reported to reflect serum concentration of the carotenoids^20,21,43,44,49–53^. Supplements induce a quick increase in serum lutein and zeaxanthin concentrations that reflect the increase in SC levels. In contrast, MP has been reported to accumulate gradually in the retina, so more time must elapse in order to detect a significant change. In other words, after taking supplements, SC levels increase first, while MP increases later.

The SC value in one subject (No.12 in Table 2) at baseline was very low. When we analyzed the SC changes without the results for this outlier subject, an increase in SC levels was still observed depending on the time period (*p* < 0.0001, two-way repeated ANOVA). The reason for the lack of significant improvement in visual functions and lack of significant correlation between contrast and/or glare sensitivity and MPOD levels/SC levels was considered to be due to good visual functions already present at baseline in healthy subjects.

Supplement intake compliance varied from 88–100%. The low supplement intake rates were due to subject forgetfulness, but no one refused to take the supplement due to change in general condition. Lutein intake of up to 1 mg/kg body weight (20 to 40 mg/day) and zeaxanthin intake of up to 0.75 mg/kg body weight are considered safe^19,54^. However, one report^55^ noted that overdose lutein resulted in yellow deposits in the retina; hence, caution is needed to avoid excessive intake of lutein. From the results of blood tests, no serious adverse effects were observed, but increase in total cholesterol and LDL-cholesterol was noted. Increase in total cholesterol at week 16 was observed in 12 subjects, of which increase from the normal range to exceeding the upper limit was observed in 4 subjects. Increase in LDL-cholesterol was observed in 11 subjects, of which increase from the normal range to exceeding the upper limit was observed in 4 subjects. The present supplement contained sunflower oil, safflower oil, fatty acid ester, and glycerol fatty acid ester. These fatty acids may influence total cholesterol and LDL-cholesterol levels. However, for three of four subjects who had total cholesterol values of higher than the normal limit at baseline, a decrease in cholesterol levels to the normal range at week 16 was observed. Cholesterol levels are assumed to be highly affected by daily diet, which was not examined in this study. Consideration of the effect of cholesterol due to the supplements is needed in the future.

From the results of this study, the effectiveness of supplements containing 20 mg of lutein and 4 mg of zeaxanthin combined with co-antioxidants on MPOV and SC levels without any serious adverse event could be demonstrated in subjects with relatively low MPOD levels at baseline. Limitations of this study included its study design as a single arm without a control group and the small number of subjects. A lack of assessment of dietary intake of lutein and zeaxanthin during the study period was another shortcoming.

## Methods

### Subjects

Sample size was based on our previous study^32^. In this study, mean MPOD was measured by the same method as the present study before and after intake of a 10 mg lutein supplement, with values of 0.62 and 0.72, respectively, with a standard deviation (SD) of 0.06. Increase was 16% which was comparable to that of other studies^30,56^. When α error is set at 0.05 and power at 0.8, the number of subjects with significant results in a comparative study was 14. Similarly, we set number of our subjects at 16. Self-selected recruitment of subjects was facilitated by intranet information of Seirei Hamamatsu General Hospital and by word of mouth. Measurements of far and near visual acuities and intraocular pressure, observation by slit-lamp biomicroscopy and fundus ophthalmoscopy, and OCT were performed before enrollment in order to assess the inclusion criteria. MPOD was measured using heterochromatic flicker photometry (HFP-MM II) with a reference point of 7° eccentricity, and subjects with a MPOD at 0.5° eccentricity of 0.64 or less were enrolled. Subjects with a MPOD greater than 0.64 were excluded. Details of the inclusion criteria are shown in Table 1. When both eyes met the inclusion criteria, the targeted eye was determined based on subject preference, and the following examinations were performed only on the targeted eye.

The study was approved by the institutional review board of Seirei Hamamatsu General Hospital (No.2965) and registered as No. UMIN000031870. All subjects provided a written informed consent form that complied with the tenets of the Declaration of Helsinki.

### Assessment of visual functions and OCT imaging

Subjects were measured for far and near visual acuities using a decimal visual acuity test chart, and given a contrast and glare sensitivity test using a contrast glare-tester (Model CGT-1000, TAKAGI, Nagano, Japan), at baseline, and 8 and 16 weeks after starting supplement intake. Contrast threshold values were assessed at six visual angles (sizes) of the target (6.3, 4.0, 2.5, 1.6, 1.0, 0.7 degrees) under mesopic (10 candelas per square meter) and glare (10,000 candelas per square meter) conditions, and the area under the log contrast sensitivity function (AULCSF) was used to evaluate contrast sensitivity in the normal setting (C_AULCSF) and in the presence of glare light (G_AULCSF). OCT imaging was obtained at baseline and 4, 8, 12, and 16 weeks after starting supplement intake. Mean retinal thickness at 1-mm diameter central area (central retinal thickness, CRT) was obtained using SPECTRALIS software by volume scan with 20° × 15° in the 19 B-scan mode.

### Measurement of macular pigment optical density

MPOD levels of the study eye were measured using two different methods at baseline and 4, 8, 12, and 16 weeks after starting supplement intake. First, MPOD was measured by HFP-MM II without mydriasis. MPOD values at 0.25° and 0.5°eccentricities with a reference point of 7° eccentricity were evaluated. Then, the pupil was dilated with 2.5% phenylephrine hydrochloride and 1% tropicamide, and MPOD was measured by two-wavelength autofluorescence imaging, AFI-SP. Basic functionality and handling of this instrument have been previously described^30,57,58^. Measurements are carried out with alternating blue and green laser light sources which are raster scanned over the retina for about 30 seconds. In contrast to earlier digital camera-based instruments that captured autofluorescence images with a single blue or green flash, AFI-SP uses a scanning laser technique that does not require pre-bleaching of photopigments. Optical densities at 0.25°, 0.5°, 1°, and 2°eccentricities (local MPODs), and total optical density volume within 9° eccentricity (total MPOV) were used for analyses. 9° eccentricity was set as a reference point. The local MPOD level is the average along a concentric circle at selected eccentricities, and total MPOV is the total of MPODs within 9° eccentricity. Total MPOV correlated to MPOD at each eccentricity and has been proposed as the preferred metric for the assessment of macular pigment^42^.

### Measurement of skin carotenoid levels

Pressure-mediated reflection spectroscopy (VEGGIE METER, Longevity Link Corporation, Salt Lake City, Utah) was used to measure SC levels. Basic functions of this device have been previously described^59^. VEGGIE METER has been validated by comparing it to skin resonance Raman spectroscopy which has high specificity for carotenoid molecules^48,60^. Calibration was performed with the provided dark and white reference materials prior to daily skin measurements. Subjects placed their left middle finger into the device’s finger cradle and pressed their fingertip against the convex contact lens surface with the assistance of a spring-loaded lid. Slight pressure was applied to the fingertip to reduce blood perfusion of the measured tissue, which prevents highly blood with its high visible light absorbance from interfering with measurement of skin carotenoid levels. SC index was determined as the average of three consecutive measurements.

### Supplement

Commercially available gelatin soft capsule supplements containing lutein/zeaxanthin (OPTIADE ML, WAKAMOTO Pharmaceutical Co., Ltd, Tokyo, Japan) were used. The subjects orally took three capsules after a meal with water once a day for 16 weeks. Contents of three capsules were: lutein 20 mg (6.7 mg per capsule), zeaxanthin (3R,3′R-zeaxanthin and 3R, 35–45%, 3′S; meso-zeaxanthin, 55–65%) 4 mg (1.3 mg per capsule), vitamin C 408 mg, vitamin E 242.3 mg, zinc 30 mg, copper 1.5 mg. Lutein and zeaxanthin material used in this supplement was Lutemax 2020 (OmniActive Health Technologies Ltd., Mumbai, India). The carotenoids were suspended in a mixture of sunflower oil, safflower oil, beeswax, fatty acid ester, and glycerol ester. Soft capsules were made from porcine gelatin and glycerol. Table 9 shows the nutritional ingredients for one day (3 capsules). Supplements were provided to each subject every four weeks. The subjects were requested to record the time the supplement was taken in a notebook each day, and any remaining capsules were collected so that the study coordinator could confirm the compliance of the subjects.

**Table 9:** Nutritional ingredients of the supplements for one day (3 capsules).

Nutritional ingredients		
Energy	(kcal)	10.7
Protein	(g)	0.59
Fat	(g)	0.64
Carbohydrate	(g)	0.64
Sodium	(mg)	0.0014

### Assessment of the safety of the supplement

Subjects were asked about their general condition and past history of illness, and measurements of height and body weight were taken at baseline. Subjects were asked to record any change in general condition, details of any medicine taken (name and amount of the drug), and any changes in their lifestyle in the notebook every day. Fasting blood and urine tests were performed at baseline and after 16 weeks. Components of the tests are described in the results. All analyses were performed by SRL Co., Ltd (Tokyo, Japan).

### Statistical analysis

Statistical analyses were performed by ORTHOMEDICO Inc. (Tokyo, Japan) using IBM SPSS Statistics version 23. Changes in numerical variables of each subject during the study period were analyzed by two-way factorial analysis of variance (ANOVA). Post-hoc analyses were performed by Fisher’s LSD or Dunnet’s test. Correlation between two numerical variables were analyzed by Pearson’s correlation coefficient test. Two average values of numerical variables were compared by t-test (two-sided). Average value was shown as the mean ± standard deviation. All statistical tests were two-sided; and significant difference was set at *p* < 0.05.

## Data Availability

The datasets generated during and/or analyzed during the current study are available from the corresponding author on reasonable request.
